# Nitric Oxide Production and Fc Receptor-Mediated Phagocytosis as Functional Readouts of Macrophage Activity upon Stimulation with Inactivated Poultry Vaccines in Vitro

**DOI:** 10.3390/vaccines8020332

**Published:** 2020-06-22

**Authors:** Robin H. G. A. van den Biggelaar, Willem van Eden, Victor P. M. G. Rutten, Christine A. Jansen

**Affiliations:** 1 Department of Biomolecular Health Sciences, Division of Infectious Diseases and Immunology, Faculty of Veterinary Medicine, Utrecht University, Yalelaan 1, 3584CL Utrecht, The Netherlands; r.h.g.a.vandenbiggelaar@uu.nl (R.H.G.A.v.d.B.); W.vanEden@uu.nl (W.v.E.); v.rutten@uu.nl (V.P.M.G.R.); 2 Department of Veterinary Tropical Diseases, Faculty of Veterinary Science, University of Pretoria, Private Bag X20, Hatfield 0028, South Africa

**Keywords:** chicken, inactivated vaccines, macrophages, HD11 cell line, phagocytosis, nitric oxide, CHIR-AB1, vaccine quality control

## Abstract

Vaccine batches must pass routine quality control to confirm that their ability to induce protection against disease is consistent with batches of proven efficacy from development studies. For poultry vaccines, these tests are often performed in laboratory chickens by vaccination-challenge trials or serological assays. The aim of this study was to investigate innate immune responses against inactivated poultry vaccines and identify candidate immune parameters for in vitro quality tests as alternatives for animal-based quality tests. For this purpose, we set up assays to measure nitric oxide production and phagocytosis by the macrophage-like cell line HD11, upon stimulation with inactivated poultry vaccines for infectious bronchitis virus (IBV), Newcastle disease virus (NDV), and egg drop syndrome virus (EDSV). In both assays, macrophages became activated after stimulation with various toll-like receptor agonists. Inactivated poultry vaccines stimulated HD11 cells to produce nitric oxide due to the presence of mineral oil adjuvant. Moreover, inactivated poultry vaccines were found to enhance Fc receptor-mediated phagocytosis due to the presence of allantoic fluid in the vaccine antigen preparations. We showed that inactivated poultry vaccines stimulated nitric oxide production and Fc receptor-mediated phagocytosis by chicken macrophages. Similar to antigen quantification methods, the cell-based assays described here can be used for future assessment of vaccine batch-to-batch consistency. The ability of the assays to determine the immunopotentiating properties of inactivated poultry vaccines provides an additional step in the replacement of current in vivo batch-release quality tests.

## 1. Introduction

Infectious diseases are a major problem for the poultry industry and lead to economic losses. Therefore, vaccination is essential to prevent disease outbreaks and maintain flock health. Early in life, starting before, or soon after hatch, chickens are vaccinated frequently with live attenuated vaccines to induce protective immunity. Before the first laying period, layers receive several booster vaccinations to maintain this protective immunity during their egg production phase. These may include inactivated multivalent vaccines against a combination of pathogens, such as infectious bronchitis virus (IBV), Newcastle disease virus (NDV), egg drop syndrome virus (EDSV), and infectious bursal disease virus (IBDV), and are often formulated as water-in-oil (*w*/*o*) emulsions using mineral oil adjuvants to potentiate the immune response [1].

Since vaccines are complex biological products, each batch requires routine quality control (QC) testing to ensure that it meets the established requirements to induce protection against disease. Quality testing for the potency of inactivated poultry vaccines used to be performed with vaccination-challenge tests, which have nowadays largely been replaced with vaccination-serology tests [2,3]. However, for many infectious diseases of poultry, the correlates of protection are still unknown and serological assays may not always be the best method to monitor vaccine potency. Moreover, there is a growing global intent to further reduce the number of laboratory animals used for routine quality control of vaccine batches [4]. As a consequence of the implementation of the 3Rs (replacement, reduction, and refinement) in European law, animal testing in the EU is only allowed when no non-animal alternatives are available [5]. The growing availability of physicochemical and cell-based alternatives has created opportunities to replace animal-based tests by methods that provide cost-effective and ethically attractive alternatives.

One strategy to ensure the quality of vaccines is the consistency approach, which implies that batch-to-batch variation of vaccines can be controlled by well-defined production processes and analysis of intermediate and final products by in vitro methods [6,7,8]. This approach requires a battery of methods to create a product profile that provides sufficient information to replace current animal-based tests. To assess antigen quantity, an important quality parameter of vaccines, a method like enzyme-linked immunosorbent assay (ELISA) can be used, which is in place for inactivated NDV vaccines [3,9,10] Moreover, such methods have been developed for inactivated IBV and IBDV vaccines [10,11], although they are not yet used for QC testing [3]. However, vaccine quality is usually not dependent on antigen quantity alone. Usually, additional vaccine components, like adjuvants, are required to stimulate the immune system, which is why antigen quantification methods should be complemented with other QC tests that assess the immunogenicity of these additives.

Pathogen-associated molecular patterns (PAMPs), like bacterial cell wall components or viral nucleic acids, activate innate immune cells via pattern recognition receptors (PRRs) [12,13]. Similarly, vaccine excipients, including adjuvants, can augment the immunogenicity of vaccines by activation of innate immune cells, either directly or by inflicting tissue damage, resulting in the release of endogenous danger signals, such as extracellular DNA, ATP, or heat-shock proteins, collectively called damage-associated molecular patterns (DAMPs) [14,15]. It is currently unknown whether inactivated viral poultry vaccines contain PAMPs or other constituents leading to DAMPs that augment immune responsiveness by the activation of innate immune cells. Immunopotentiating effects of vaccine constituents on innate immune cells can be addressed in vitro with cell-based assays. For poultry vaccines, a good candidate for such assays is the chicken macrophage-like cell line HD11, which shows broad expression of PRRs, including toll-like receptors (TLRs) [16]. The HD11 cell line has already been employed to explore the immune-activating or immunomodulatory properties of TLR agonists [17,18,19], liposomes [20], host defense peptides [21], cytokines [22,23,24], bacteria [17,21,25], and replicating viruses [26,27,28], using nitric oxide production as a functional readout of macrophage activation. Macrophages produce nitric oxide using the enzyme-inducible nitric oxide synthase (iNOS), which is expressed upon stimulation of PRRs and downstream activation of transcription factor nuclear factor kappa B (NF-κB) [17]. In addition, the HD11 macrophage-like cell line expresses a high-affinity Fc receptor for immunoglobulin Y (IgY), the chicken Ig-like receptor AB1 (CHIR-AB1) [29], and has been used before to study phagocytosis [30,31]. In vivo, macrophages are strategically located at the entry sites of lymphoid tissues and are able to react to PAMPs and DAMPs in the blood lymph fluid [32,33]. Moreover, macrophages have been shown to augment immune responses after vaccination, due to their ability to produce nitric oxide and perform Fc receptor-mediated phagocytosis [34,35,36].

In this study, we stimulated the chicken macrophage-like cell line HD11 with inactivated poultry vaccines and intermediary vaccine products, including antigenic fractions and mineral oil adjuvant, to study their effects on nitric oxide production and phagocytosis and to contribute to a better understanding of their effects on the chicken innate immune system. Moreover, we explored the potential of nitric oxide production and phagocytosis as biomarkers of vaccine-induced immune activation, for future use in in vitro vaccine QC tests. 

## 2. Materials and Methods 

### 2.1. HD11 Cell Culture and Stimulation

The chicken macrophage-like cell line HD11 [37], stored at −140 °C in complete Roswell Park Memorial Institute (RPMI)-1640 medium with 50% Fetal Bovine Serum (FBS) and 10% dimethyl sulfoxide (DMSO), was thawed and used after 3 to 20 passages. The cells were maintained in complete RPMI-1640 cell culture medium supplemented with GlutaMAX-I, phenol red, HEPES, 10% fetal bovine serum (FBS), 200 U/mL penicillin, and 200 U/mL streptomycin (all Gibco, Life Technologies Limited, Paisley, UK) in Corning 75-cm^2^ cell culture flasks (Sigma-Aldrich, Saint Louis, MO, USA) at 37 °C, 5% CO_2_, and passaged twice weekly. For experiments, HD11 cells were harvested from 75-cm^2^ cell culture flasks when they were at ~90% confluency using a 0.25% trypsin/EDTA solution supplemented with phenol red (Gibco, Life Technologies Limited, Paisley, UK). Subsequently, the cells were counted and resuspended at a concentration of 200,000 cells/mL. The cells were seeded at 1 mL/well complete RPMI medium in Corning Costar 24-well cell culture plates (Sigma-Aldrich, Saint Louis, MO, USA) and cultured overnight at 37 °C and 5% CO_2_. 

After overnight incubation, the HD11 cells were exposed to various stimuli to assess their activation using either nitric oxide production or phagocytosis as a readout. Stimuli included 100–300 ng/mL lipopolysaccharides (LPSs) from *E. coli* O127:B8 (Sigma-Aldrich, Saint Louis, MO, USA) to target TLR4, 100–500 ng/mL CpG oligonucleotides (ODNs) 2006 to target TLR21, 10 μg/mL resiquimod (R848) to target TLR7, 10 ng/mL Pam3CSK4 to target the TLR2/1 heterodimer, and 5 μg/mL zymosan from *S. cerevisiae* (all InvivoGen, San Diego, CA, USA) to target the TLR2/6 heterodimer. In addition, HD11 cells were stimulated with established inactivated poultry vaccines and/or their antigenic fractions, which were kindly provided by three pharmaceutical companies that are part of the VAC2VAC consortium (http://www.vac2vac.eu/), hereafter referred to as companies A, B, and C. The inactivated poultry vaccines used in this study contained mineral oil adjuvants in *w*/*o* formulation and included inactivated monovalent IBV (company B), inactivated bivalent IBV + NDV (companies A, B, and C), and inactivated trivalent IBV + NDV + EDSV (company A) vaccines. The inactivated poultry vaccines from companies A, B, and C were prepared in such a way that a single chicken vaccination dose corresponds to, respectively, 0.5, 0.5, and 0.3 mL. The antigenic fractions, hereafter referred to as antigens, comprised whole inactivated IBV (companies A, B, and C) and NDV (company B), which were propagated on embryonated chicken eggs, harvested from the allantoic cavity, and inactivated using either formaldehyde or β-propiolactone. Allantoic fluid without virus (company A), mineral oil (company A), and an “empty vaccine” consisting of allantoic fluid without virus formulated with mineral oil (company B) were included as controls

### 2.2. Griess Assay to Measure Nitric Oxide Production by HD11 Cells 

Nitric oxide production by HD11 cells was measured by the Griess assay [38] 48 h after stimulation. First, 50 μL of supernatants were harvested from triplicate wells and transferred to a 96-well flat-bottom plate (Corning B.V. Life Sciences, Amsterdam, The Netherlands) to measure the nitrite concentration. A 3.13–200 μM NaNO_2_ nitrite standard dilution series (Sigma-Aldrich, Merck, St. Louis, MO, USA) was included to generate a standard curve. Griess assay reagents were made by dissolving N-(1-naphtyl)ethylenediamine at 3 g/L and sulfanilamide at 10 g/L (both from Sigma-Aldrich, Merck, St. Louis, MO, USA) in 2.5% phosphoric acid (Supelco, Merck, St. Louis, MO, USA). The Griess reagents were mixed 1:1 and 50 μL was added to the wells with cell culture supernatants and standards. The Griess reagents mixture turned purple upon reaction with nitrite ions in the cell culture supernatant. The optical density (OD) at 540 nm of each well was measured using a FLUOstar Omega microplate reader (BMG Labtech, Ortenberg, Germany) to determine the nitrite concentration of each sample according to the nitrite standard curve. 

### 2.3. Phagocytosis of IgY-Opsonized Beads by HD11 Cells

#### 2.3.1. IgY-Opsonization of Fluorescent Beads

The phagocytosis assay was performed with chicken IgY-opsonized fluorescent beads, which were prepared by mixing 1 μm crimson carboxylate-modified FluoSpheres (Invitrogen, Life Technologies Europe BV, Bleiswijk, The Netherlands) at a final concentration of 7.2 × 10^9^/mL with an egg yolk IgY fraction (Agrisera AB, Vännäs, Sweden) at a final concentration of 14.4 mg/mL in a glass tube, followed by overnight mixing in an orbital rotator at 4 °C. The next day, the beads were washed twice by adding 10 mL Dulbecco’s phosphate-buffered saline without calcium and magnesium (DPBS^−/−^; Lonza, Basel, Switzerland) and centrifugated at 3000× *g* for 20 min between washes. Finally, the beads were resuspended in DPBS^−/−^ at a concentration of 3.5 × 10^9^ beads/mL. Coupling of IgY was confirmed by staining the beads with 0.5 μg/mL R-phycoerythrin (PE)-labeled mouse anti-chicken monoclonal antibodies (SouthernBiotech, Birmingham, AL, USA) in fluorescence-activated cell sorting (FACS) buffer containing DPBS^−/−^ + 0.5% bovine serum albumin and 0.005% sodium azide (both from Sigma-Aldrich, Saint Louis, MO, USA) and analysis using a CytoFLEX LX flow cytometer and 375-, 561-, and 638-nm lasers (Beckman Coulter Inc., Brea, CA, USA) (Supplementary Materials Figure S1).

#### 2.3.2. Phagocytosis by HD11 Cells

Phagocytosis of IgY-opsonized beads was measured 24 h after stimulation of the HD11 cells. First, three wells of a 24-well plate with HD11 cells were harvested using DPBS^−/−^ supplemented with 5 mM UltraPure EDTA (Invitrogen, Life Technologies Europe BV, Bleiswijk, The Netherlands) to determine the cell counts per well, which ranged between 0.5 and 1.0 × 10^6^ cells. IgY-opsonized beads were added at a 1:1 bead-to-cell ratio to the HD11 cells of the remaining wells followed by a 4-h incubation at 37 °C, 5% CO_2_ to allow the cells to phagocytose the beads. Next, the cells were harvested using DPBS^−/−^ supplemented with 5 mM UltraPure EDTA and centrifuged at 400× *g* for 3 min. The cells were transferred to 96-well V-bottom plates (Greiner Bio-One B.V., Alphen aan den Rijn, The Netherlands), washed in DPBS^−/−^, and stained for cell viability in 50 μL DPBS^−/−^ with 1:400 Zombie Aqua Fixable Viability Dye (BioLegend Inc., San Diego, CA, USA) for 20 min at 4 °C. Subsequently, the cells were washed twice and fixed in 200 μL DPBS^−/−^ with 2% paraformaldehyde (Alfa Aesar, Haverhill, MA, USA) for 10 min at room temperature (RT). Finally, the cells were washed once more in FACS buffer and resuspended in 200 μL of FACS buffer. Up to 50,000 cells were analyzed using the CytoFLEX LX flow cytometer. 

**Figure 1:**
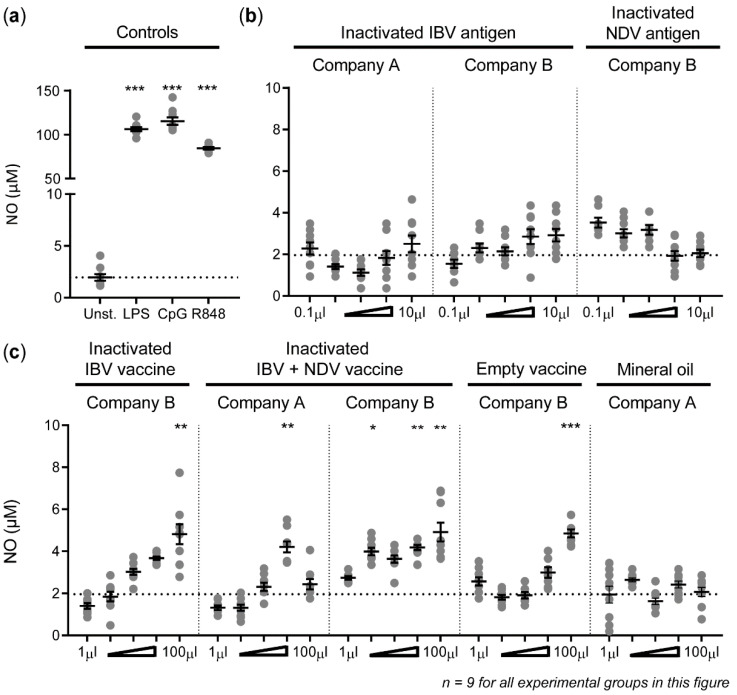
Inactivated poultry vaccines induced nitric oxide production by HD11 cells, whereas inactivated IBV and NDV antigens did not. (**a**) Nitric oxide production by HD11 cells was assessed upon stimulation with TLR agonists, i.e., 100 ng/mL LPS, 100 ng/mL CpG, and 10 μg/mL R848. (**b**) In addition, HD11 cells were exposed to inactivated IBV and NDV antigens (companies A and B) in doses ranging from 0.1–10 μL/mL. (**c**) Finally, HD11 cells were exposed to vaccines, an “empty vaccine” containing allantoic fluid without inactivated viruses, and mineral oil in doses ranging from 1–100 μL/mL. Three independent experiments were performed, and the experimental conditions of each independent experiment were tested in triplicate. Error bars represent the standard error of the mean (SEM). The experimental groups were tested for statistically significant increases in nitric oxide production as compared to unstimulated HD11 cells using a Kruskal–Wallis test and Dunn’s multiple comparisons test. Statistical significance is indicated by * *p* < 0.05, ** *p* < 0.01, and *** *p* < 0.001.

**Figure 2:**
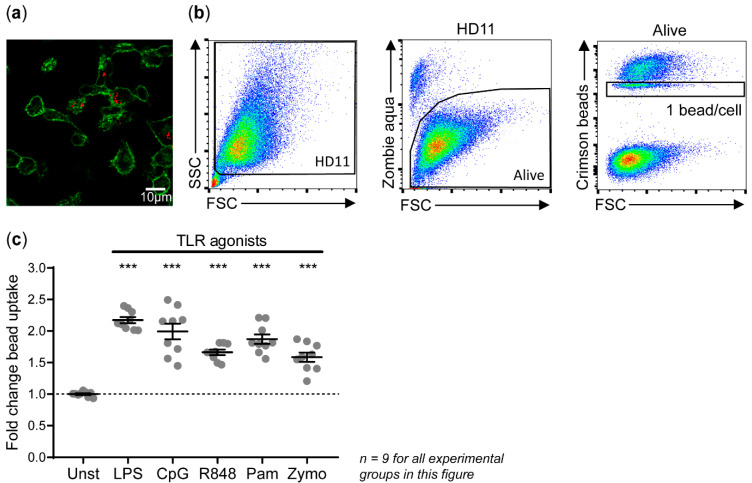
TLR agonists stimulated the uptake of IgY-opsonized beads by HD11 cells. (**a**) Confocal microscopy confirmed the uptake of IgY-opsonized beads by HD11 cells. The surface of unstimulated HD11 cells was made visible by WGA-Alexa Fluor 488 shown in green and IgY-opsonized beads are shown in red. A corresponding video showing the 3-D model of this composition can be found in Supplementary Materials Video S1. (**b**) Bead uptake by HD11 cells was quantified by flow cytometry. HD11 cells were gated for their scatter profile (FSC/SSC) and viability (zombie aqua live/dead staining). Moreover, HD11 cells with 1 bead/cell were gated to determine the fluorescence of a single bead, from which the average beads/cell for all HD11 cells could be calculated. (**c**) HD11 cells were stimulated with 300 ng/mL LPS, 500 ng/mL CpG, 10 µg/mL R848, 10 ng/mL Pam3CSK4 (Pam), 5 µg/mL zymosan (Zymo), or left unstimulated (Unst). The results are expressed as fold changes in bead uptake after stimulation in comparison to unstimulated controls. Three independent experiments were performed, and the experimental conditions of each independent experiment were tested in triplicate. Error bars represent the standard error of the mean (SEM). The experimental groups were tested for statistically significant differences in bead uptake between stimulated and unstimulated groups using a one-way ANOVA and Holm–Sidak’s multiple comparisons test. Statistical significance is indicated by *** *p* < 0.001.

Data analysis was performed using FlowJo Software v. 10.6 (FlowJo LCC, Ashland, OR, USA) and Prism 8.4 (Graphpad Software Inc., San Diego, CA, USA). The viability of the cells was expressed as the percentage of HD11 cells negative for Zombie Aqua Fixable Viability Dye. Only samples with ≥100 viable cells in the live gate (Figure 2b) were included in the analysis of changes in bead uptake. The fluorescent content of HD11 cells measured using a 660/10-nm bandpass filter after excitation with the 638-nm laser was directly proportional to the number of beads engulfed. Furthermore, the HD11 containing a single bead were visible as the first positive peak in a histogram showing the fluorescent intensity at 660/10 nm. From this, the average bead uptake by each sample was calculated by:

(1) $$beads/cell=\frac{MFI_{total\;}}{MFI_{1\;bead/cell}},$$

with *MFI_total_* for the mean fluorescent intensity (MFI) at 660/10 nm of each sample *MFI*_1 *bead/cell*_ for the MFI at 660/10 nm of cells containing 1 bead/cell (see also Figure 1a). Next, the fold change in bead uptake by HD11 cells after stimulation was calculated by:

(2) $$foldchange=\frac{beads/cell_{stimulated\;}}{beads/cell_{unstimulated}}.$$

#### 2.3.3. Flow Cytometric Side Scatter to Determine Vaccine Decomposition

An accumulation of vacuoles was observed in HD11 cells exposed to inactivated poultry vaccines or the empty vaccine control (without viral antigens), which was captured by light microscopy using an an EVOS FL microscope (AMG, Mill Creek, Washington, DC, USA). The vacuoles were considered to be vaccine-derived lipid droplets, since they were only observed in the presence of inactivated poultry vaccines containing emulsified mineral oil adjuvant. The accumulation of lipid droplets was considered as a surrogate marker for vaccine decomposition and quantified by measuring the average side scatter (SSC) of HD11 cells by flow cytometry, in accordance with a previous study [39].

#### 2.3.4. Involvement of the Fc Receptor CHIR-AB1 in IgY-Opsonized Bead Uptake by HD11 Cells

HD11 cells stimulated for 24 h with different concentrations of LPS or inactivated IBV antigen (company B) were assessed for expression of CHIR-AB1 by subsequently staining the cells in 50 μL of FACS buffer with 1:20-diluted hybridoma supernatant containing mouse-anti-chicken CHIR-AB1 (clone 8D12, mouse IgG2b, gift from Thomas W. Göbel, LMU Munich, Munich, Germany) and 0.1 μg/mL allophycocyanin (APC)-labeled goat-anti-mouse IgG2a (SouthernBiotech, Birmingham, AL, USA) for 20 min at 4 °C, with two washing steps in FACS buffer in between. Next, the cells were washed once in FACS buffer and once in DPBS^−/−^ followed by staining in 50 μL of DPBS^−/−^ with 1:400 Zombie Aqua Fixable Viability Dye (BioLegend Inc., San Diego, CA, USA) for 20 min at 4 °C. Finally, the cells were washed once more in FACS buffer and resuspended in 200 μL of FACS buffer for analysis using the CytoFLEX LX flow cytometer.

To determine the involvement of CHIR-AB1 in the uptake of IgY-opsonized beads, different concentrations of mouse-anti-chicken CHIR-AB1 were administered to HD11 cells 10 min before addition of the beads to block interactions between CHIR-AB1 and IgY-opsonized beads. Subsequent steps were according to the phagocytosis assay as described.

### 2.4. Confocal Microscopy to Assess Internalization of IgY-Opsonized Beads by HD11 Cells

HD11 cells were prepared for confocal microscopy to confirm the internalization of IgY-opsonized beads. Ethanol-cleaned 12-mm glass coverslips (Waldemar Knittel Glasbearbeitungs GmbH, Brunswick, Germany) were added to the 24-well cell culture plates before HD11 cells were seeded and subjected to the phagocytosis assay as described. After 4 h of incubation with IgY-beads, the cells were washed twice with cold DPBS with calcium and magnesium (DPBS^+/+^; Lonza, Basel, Switzerland) before staining in DPBS^+/+^ with 2 μg/mL wheat germ agglutinin (WGA)-Alexa Fluor 488 (Invitrogen, Life Technologies Europe BV, Bleiswijk, The Netherlands) for 20 min at 4 °C. Subsequently, the cells were washed thrice with cold DPBS^+/+^ and fixed in DPBS^+/+^ with 4% paraformaldehyde at RT for 30 min. Next, the cells were washed three times with DPBS^−/−^ + 10 mM glycine (Merck Millipore, Burlington, MA, USA) to quench the remaining paraformaldehyde. The cells were washed once more in distilled water before the coverslips with HD11 cells were mounted on Polysine microscope slides (Menzel Glazer GmbH & Co KG, Braunschweig, Germany) using Fluorsave Reagent (Calbiochem, Merck Millipore, Burlington, MA, USA). The cells were captured, and bead internalization was analyzed using a TCS-SPE-II confocal microscope (Leica Microsystems B.V., Amsterdam, The Netherlands) and 488- and 635-nm diode lasers. Microscopic images were further processed using Fiji software [40].

### 2.5. Statistical Analysis

Statistical analysis was performed using GraphPad Prism 8.4 software. When the assumptions of normally distributed data and residuals were met, a one-way ANOVA with Holm–Sidak’s multiple comparisons test was used to test for statistically significant differences between stimulated and unstimulated control samples. When the assumptions of normality were not met, a non-parametric Kruskal–Wallis test with Dunn’s multiple comparisons test was used instead. A *p*-value of <0.05 was considered statistically significant. 

## 3. Results

### 3.1. High Concentrations of Inactivated Poultry Vaccines Induce Nitric Oxide Production by HD11 Cells

Production of nitric oxide by HD11 cells stimulated with TLR agonists, used as positive controls, was determined by the Griess assay. Stimulation with LPS resulted in 106.1 ± 2.2 μM, CpG in 115.2 ± 4.2 μM, and R848 in 84.4 ± 1.4 μM nitric oxide in the culture supernatant (Figure 1a). Stimulation of HD11 cells with dose ranges of inactivated IBV and NDV antigens provided by different companies did not result in nitric oxide production (Figure 1b). In contrast, nitric oxide was produced at low quantities when HD11 cells were stimulated with an inactivated monovalent IBV vaccine from company B (4.8 ± 0.5 μM) and inactivated bivalent IBV + NDV vaccines from companies A (4.2 ± 0.3 μM) and B (4.9 ± 0.5 μM) (Figure 1c). An “empty vaccine” without inactivated viral antigens also induced the production of low quantities of nitric oxide (4.9 ± 0.2 μM). All vaccines contained a mineral oil adjuvant and were formulated as water-in-oil emulsions. Nitric oxide production was not significantly different from unstimulated HD11 cells when non-emulsified mineral oil was added to the HD11 cells (respictively 2.0 ± 0.3 μM and 2.6 ± 0.1 μM). Taken together, small amounts of nitric oxide were produced by HD11 cells upon exposure to the inactivated poultry vaccines, which may be induced by the presence of emulsified mineral oil. 

### 3.2. Phagocytosis of IgY-Opsonized Beads by HD11 Cells Is Enhanced upon Stimulation with TLR Agonists

The ability of HD11 cells to phagocytose IgY-opsonized beads after 24 h of stimulation with TLR agonists was assessed by a 4-h co-incubation (Figure 2a and Supplementary Materials Video S1). HD11 cells showed increased uptake of IgY-opsonized beads upon stimulation with LPS (2.18 ± 0.05-fold), CpG (1.99 ± 0.12-fold), R848 (1.66 ± 0.04-fold), Pam3CSK4 (1.87 ± 0.08-fold), and zymosan (1.59 ± 0.07-fold) compared to unstimulated cells (Figure 2b,c). The viability of HD11 remained unaffected by stimulation with zymosan and was only slightly affected by LPS, CpG, R848, or Pam3CSK4 (Supplementary Materials Figure S2).

### 3.3. Allantoic Fluid-Containing Inactivated IBV and NDV Antigens Enhance Phagocytosis by HD11 Cells

**Figure 3:**
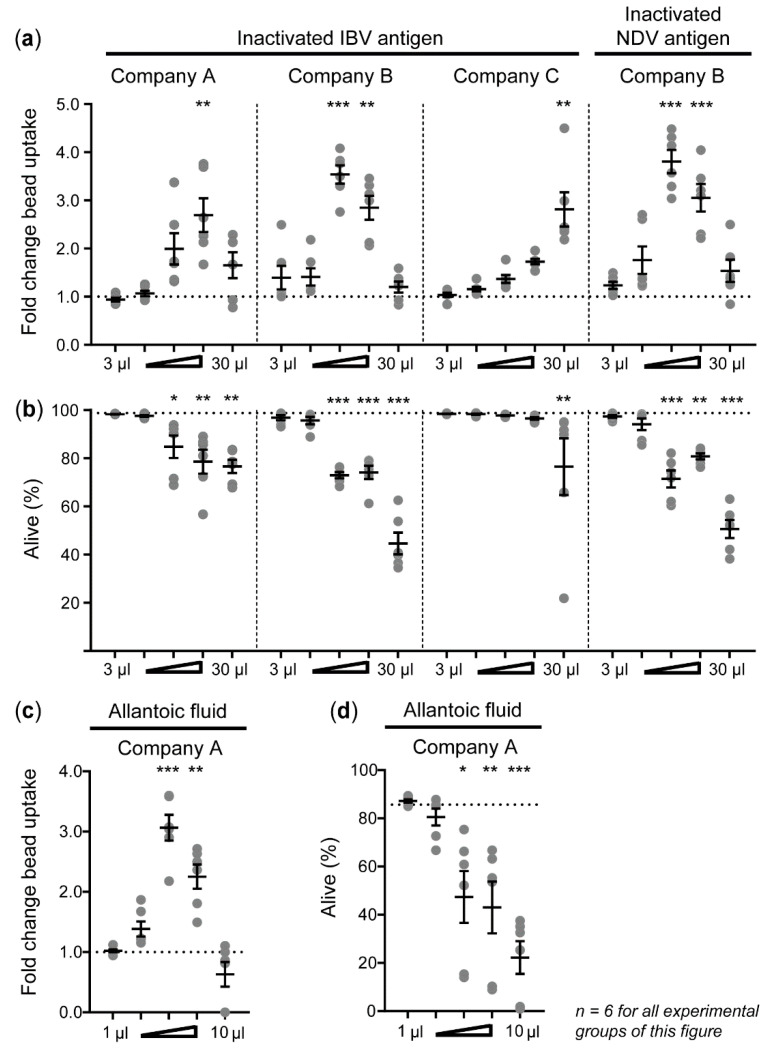
Phagocytosis of IgY-opsonized beads by HD11 cells is increased upon exposure to inactivated IBV and NDV antigens. (**a**) Differences in bead uptake upon exposure to IBV and NDV antigens are expressed as fold changes compared to unstimulated controls. (**b**) The effects of IBV and NDV antigens on HD11 cell viability, as determined by Zombie Aqua Fixable Viability Dye, is expressed as the percentage of living cells (% alive). Inactivated IBV antigens were provided by three different companies (A–C) and inactivated NDV antigen was provided by one company (B). In addition, the effects of allantoic fluid without virus (provided by company A) on HD11 cell phagocytosis capacity (**c**) and cell viability (**d**) were determined. The *x*-axis shows the titrated doses at which IBV antigens, NDV antigens, or allantoic fluid without antigens were added, expressed as μL dose, added to 1 mL of cell culture medium. Three independent experiments were performed, and the experimental conditions of each independent experiment were tested in duplicate. Error bars represent the SEM. The experimental groups were tested for statistically significant differences in bead uptake and viability between stimulated and unstimulated groups using Kruskal–Wallis tests and Dunn’s multiple comparisons tests. Statistical significance is indicated by * *p* < 0.05, ** *p* < 0.01, and *** *p* < 0.001.

Next, the effects of inactivated IBV and NDV antigens on phagocytosis by HD11 cells were determined. IBV antigen from company B (maximum fold change 3.54 ± 0.19 at 10 μL/mL) led to a higher induction of bead uptake, at a lower dose, than IBV antigens from companies A (maximum fold change 2.69 ± 0.35 at 18 μL/mL) or C (maximum fold change 2.81 ± 0.36 at 30 μL/mL) (Figure 3a). Inactivated NDV antigens from company B (maximum fold change 3.81 ± 0.29 at 10 μL/mL) also enhanced phagocytosis by HD11 cells. Beyond the doses inducing a maximum bead uptake, increasing doses of IBV and NDV antigen led to a decrease in phagocytosis (Figure 3a), concurrent with decreased cell viability (Figure 3b). 

IBV and NDV antigens both contained viruses that were whole inactivated after propagation in embryonated chicken eggs and harvest from the allantoic cavity. For this reason, allantoic fluid from non-inoculated eggs was tested for its ability to stimulate phagocytosis and found to enhance phagocytosis by 3.06 ± 0.21-fold (Figure 3c). Similar to the IBV and NDV antigens, increasing doses of allantoic fluid also led to a decrease in cell viability (Figure 3d).

### 3.4. Inactivated IBV and NDV Vaccines Can Enhance Phagocytosis by HD11 Cells 

**Figure 4:**
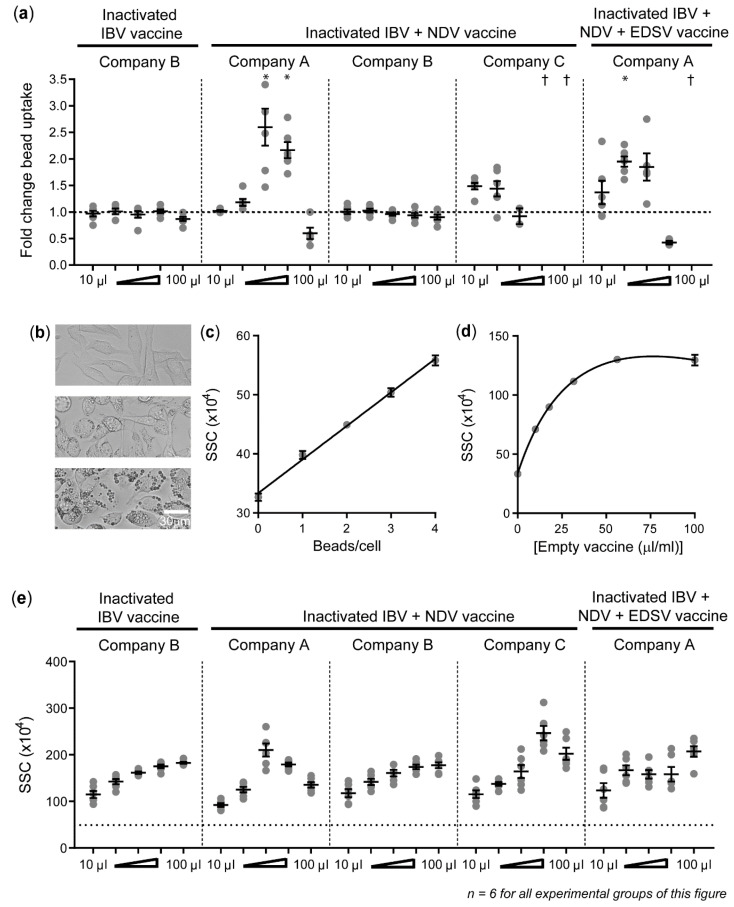
Phagocytosis capacity of HD11 cells can be increased upon exposure to inactivated viral *w*/*o* vaccines. (**a**) The fold change in bead uptake by HD11 cells upon stimulation with vaccines is compared to unstimulated controls. The *x*-axis shows the graded doses at which the vaccines have been added, expressed as μL dose, added to 1 mL of cell culture medium. † indicates that datapoints were missing because the threshold of ≥100 viable cells was not reached. (b) Light microscopy photos show unstimulated HD11 cells (top), HD11 cells exposed to 10 μL/mL inactivated bivalent vaccine B (middle), and 100 μL/mL inactivated bivalent vaccine B (top). (c) A linear correlation curve shows the relationship between the average flow cytometric SSC and number of IgY-opsonized beads/cell for HD11 cells containing 0–4 beads/cell. (d) A non-linear saturation curve shows the relationship between the average SSC and different doses of empty vaccine (without viral antigens) from company B. (e) The flow cytometric SSC of HD11 cells is shown for graded doses of the different vaccines. Three independent experiments were performed, and the experimental conditions of each independent experiment were tested in duplicate. Error bars represent the SEM. The experimental groups were tested for statistically significant differences in bead uptake and SSC between stimulated and unstimulated groups using Kruskal–Wallis tests and Dunn’s multiple comparisons tests. Statistical significance is indicated by * *p* < 0.05. For figure (e), all data was found to be statistically different from the unstimulated sample with *p* < 0.001.

Five inactivated vaccines, all containing a mineral oil adjuvant in a *w*/*o* formulation, were used to stimulate HD11 cells and included a monovalent IBV vaccine (company B), three bivalent IBV + NDV vaccines (companies A, B, and C), and a trivalent IBV + NDV + EDSV vaccine (company A). Both bi- (2.60 ± 0.35-fold) and trivalent (1.95 ± 0.10-fold) vaccines from company A led to a clear increase in the uptake of IgY-opsonized beads (Figure 4a). The bivalent vaccine from company C resulted in maximal uptake at 10μL/mL (1.49 ± 0.06). The vaccines from company B did not enhance phagocytosis. Vaccine doses beyond those inducing a maximum increase of phagocytosis resulted in decreased bead uptake, concurrent with decreased cell viability (Figure 4a and Supplementary Materials Figure S3). An increasing dose of bivalent vaccine from company B also led to a decrease in cell viability without any enhancement of phagocytosis.

The mineral oil adjuvant of the vaccines acts as a slowly decomposing depot [41], resulting in a gradual release of vaccine components, such as antigen, allantoic fluid, and mineral oil. We hypothesized that the decomposition rate of an emulsified vaccine affects its release of allantoic fluid and thus its effect on phagocytosis. Light microscopy showed the intracellular accumulation of vacuoles in HD11 cells exposed to the vaccines (Figure 4b), suggesting that HD11 cells engulfed emulsified mineral oil released by the decomposing vaccines and stored this into lipid droplets. Because of this finding, we aimed to use flow cytometric SSC to quantify the accumulation of lipid droplets as a readout for vaccine decomposition. SSC can be used as a readout for engulfed particle content, as demonstrated by the high correlation between the SSC and the number of fluorescent IgY beads after the phagocytosis assay in unstimulated HD11 cells (r^2^ = 0.996) (Figure 4c). Next, SSC was found to become higher with increasing doses of the empty vaccine control from company B until saturation was reached (r^2^ = 0.972) (Figure 4d), demonstrating that SSC could indeed be used to quantify vaccine decomposition. The SSC of HD11 cells upon exposure to the vaccines was analyzed to determine whether there was a relationship between the vaccine decomposition rate and bead uptake. For the bi- and trivalent vaccines from company A, bead uptake increased simultaneously with SSC, suggesting that the increase in bead uptake correlated with vaccine decomposition (Figure 4a,e). HD11 cells were saturated with lipid droplets after exposure to 30 μL/mL bivalent vaccine from company A, which is the dose at which phagocytosis was found to be maximally enhanced (Figure 4a,e). The SSC also increased with increasing concentrations of the mono- and bivalent vaccines from companies B and C, but this did not result in similar changes in bead uptake. This indicates that differences between the vaccines in their capacity to induce phagocytosis cannot solely be explained by different decomposition rates.

### 3.5. Fc Receptor CHIR-AB1 Is Responsible for the Enhancement of IgY-Opsonized Bead Uptake by HD11 Cells upon Exposure to Inactivated IBV Antigen 

**Figure 5:**
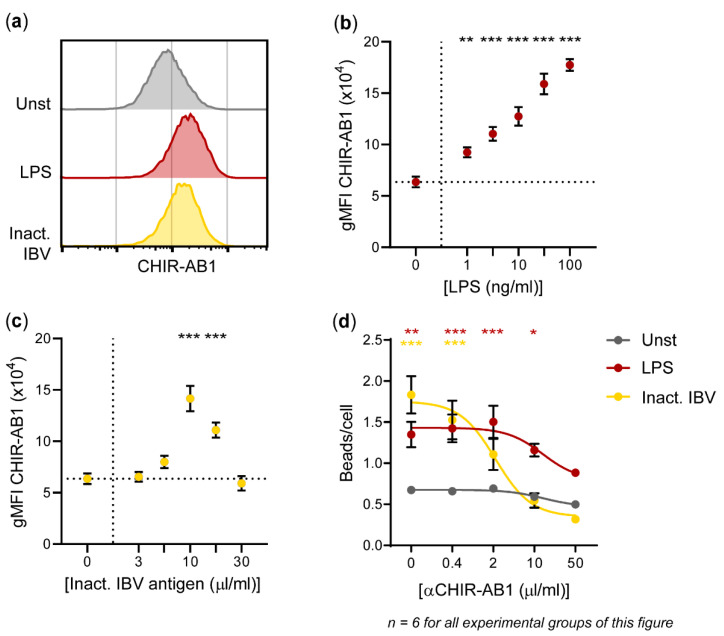
The induction of phagocytosis after exposure to inactivated IBV antigen is dependent on the IgY Fc receptor CHIR-AB1. (**a**) Representative histograms show CHIR-AB1 expression by HD11 cells after 24 h without stimulation, stimulation with 100 ng/mL LPS, or stimulation with 10 μL/mL IBV antigen. (**b**,**c**) CHIR-AB1 surface expression by HD11 cells was quantified and expressed as the geometric mean fluorescent intensity (gMFI) after 24 h stimulation with different concentrations of LPS (**b**) and IBV antigen (**c**). (**d**) Unstimulated HD11 cells and HD11 cells stimulated with LPS or inactivated IBV antigen for 24 h received the blocking antibody 8D12 specific for chicken CHIR-AB1 10 min before the addition of IgY-opsonized beads. The average number of phagocytosed beads per HD11 cell is shown for different concentrations of blocking antibody. Three independent experiments were performed, and the experimental conditions of each independent experiment were tested in duplicate. Error bars represent the SEM. The experimental groups were tested for statistically significant differences in CHIR-AB1 expression or bead uptake between stimulated and unstimulated groups using one-way ANOVA tests and Holm–Sidak’s multiple comparisons tests. Statistical significance is indicated by * *p* < 0.05, ** *p* < 0.01, and *** *p* < 0.001.

Since we chose to use IgY-opsonized beads, we investigated whether the increase in phagocytosis was dependent on the high-affinity IgY Fc receptor CHIR-AB1. Its expression increased upon 24 h of stimulation with LPS (maximum 2.79 ± 0.09-fold change in gMFI) or inactivated IBV antigen (maximum 2.23 ± 0.19-fold change in gMFI) (Figure 5a,b). For IBV antigen, CHIR-AB1 expression reached its peak after stimulation with a dose of 10 μL/mL and decreased at higher doses, concurrent with the previously described cytotoxicity of the antigen (Figure 3b). Next, HD11 cells were incubated with a CHIR-AB1 blocking antibody before performing the phagocytosis assay. The increased bead uptake upon exposure to an IBV antigen diminished with increasing concentrations of the blocking antibody (from 1.83 ± 0.23 beads/cell to 0.32 ± 0.04 beads/cell) (Figure 5d). In contrast, bead uptake in unstimulated or LPS-stimulated HD11 cells was less affected by CHIR-AB1 blocking (Unst: from 0.67 ± 0.05 beads/cell to 0.50 ± 0.04 beads/cell; LPS: from 1.35 ± 0.15 beads/cell to 0.89 ± 0.05 beads/cell). 

## 4. Discussion

In the present study, putative activating properties of inactivated poultry vaccines and their constituents on innate immune cells were assessed through measurement of nitric oxide production and phagocytosis by the chicken macrophage-like cell line HD11, as potential biomarkers for in vitro assessment of batch-to-batch consistency of vaccines. Small amounts of nitric oxide were produced in the presence of inactivated IBV and NDV vaccines or an empty vaccine without viral antigens. In contrast, nitric oxide was not produced in the presence of inactivated IBV or NDV antigens or non-emulsified mineral oil. These findings indicate that nitric oxide was produced by HD11 cells due to stimulation with the mineral oil adjuvant in its emulsified form. 

The absence of nitric oxide production in the presence of viral antigens was unexpected, since HD11 cells do express TLR7 [42] and TLR21 [43], PRRs for viral single-stranded RNA and double-stranded DNA, respectively. Moreover, TLR7 agonist R848 and TLR21 agonist CpG were able to induce high amounts of nitric oxide production. One hypothesis is that the nucleic acids from the inactivated viral antigens did not end up in the endosomes where these TLRs are present [42]. Alternatively, the recognition of the viral nucleic acids could be impaired by chemical modification, as a result of viral inactivation, or degradation. It has been shown for H5N1 influenza vaccines that TLR7 is involved in the generation of an effective adaptive immune response and that TLR7 activation is severely reduced after inactivation of the antigen with formalin or β-propiolactone [44,45].

The phagocytosis assays showed that both inactivated poultry vaccines and antigens enhanced the phagocytosis of IgY-opsonized beads by HD11 cells. Moreover, allantoic fluid without antigens stimulated HD11 cells to enhance phagocytosis, similar to allantoic fluid containing inactivated IBV and NDV antigens. These results indicate that allantoic fluid, present in both the vaccines and antigen preparations, was responsible for the enhancement of phagocytosis. Allantois fluid contains a high concentration of uric acid, which may form monosodium urate crystals [46,47]. Previous studies have shown that uric acid is released from cells damaged by aluminum salt adjuvants and may form monosodium urate crystals that act as immunostimulatory DAMPs [47]. The stimulatory effects of allantoic fluid on phagocytosis may therefore be caused by the presence of uric acid precipitates. Vaccination studies in mice have shown that DAMPs stimulate antigen uptake by macrophages in vitro and enhance antibody titers in vivo [48,49,50]. Whether allantoic fluid stimulates phagocytosis and enhances antibody titers in chickens in vivo will be of interest for further investigation. 

Stimulation with inactivated IBV antigens from different sources induced different outcomes of the phagocytosis assay, both in terms of the dose at which the highest bead uptake was observed and the maximal bead uptake, whereas no differences were observed between IBV and NDV antigens from the same source. Similarly, vaccines from different courses resulted in different outcomes. However, this does not affect the feasibility of assessing vaccine batch quality under the consistency approach, which aims to select a number of parameters for individual vaccines or intermediate products to prove batch-to-batch consistency.

Besides antigens, the inactivated poultry vaccines in this study contained mineral oil adjuvants in *w*/*o* formulation, which has been shown to form a depot with a low decomposition rate in vivo, leading to slow release kinetics of the antigen [51,52]. In our study, some vaccines enhanced bead uptake by HD11 cells, whereas other vaccines did not, depending on the manufacturer of the vaccines. These results may be explained by differences in the antigen dose or release kinetics of the different products, which depends on the product ingredients and the manufacturing process. Light microscopy showed the accumulation of lipid droplets in HD11 cells upon stimulation with emulsified mineral oil or vaccines containing emulsified mineral oil. We used flow cytometric SSC, which measured cell contents and granularity [39], to assess quantitative differences in the accumulation of lipid droplets as a read-out for decomposition of the vaccines. Although, SSC increased upon incubation with higher concentrations of the individual vaccines, this did not always result in increased bead uptake. This suggests that the variation in phagocytosis between the vaccines cannot solely be explained by differences in decomposition rates. Therefore, phagocytosis may be affected by other determinants of the vaccine formulation, including differences in antigen content, the immunostimulatory capacity of the allantoic fluid, or cytotoxicity.

Since CHIR-AB1 is known as a high-affinity chicken IgY Fc receptor that signals through Fc ε receptor I gamma chain (FcεRIγ) upon interaction with heat-aggregated IgY [29], we investigated its involvement in the uptake of IgY-opsonized beads by HD11 cells upon stimulation. We observed increased CHIR-AB1 expression on HD11 cells upon stimulation with LPS and inactivated IBV antigen. In addition, blocking CHIR-AB1 diminished the increase in phagocytosis as a result of stimulation. Hence, it must be concluded that inactivated IBV antigen led to an increase in phagocytosis due to an increase in the expression of CHIR-AB1. The increase in phagocytosis was therefore Fc receptor dependent. In a previous study, LPS was not found to affect CHIR-AB1 expression by primary macrophages, which contrasts to our findings in the HD11 cell line [29]. The difference may also be explained by the use of another type of LPS.

The nitric oxide production assay and the phagocytosis assay presented in this study showed different levels of macrophage activity upon stimulation with individual inactivated poultry vaccines. Based on our results, nitric oxide production by HD11 cells seemed to be induced by the mineral oil adjuvant, when present in a water-in-oil formulation. Fc receptor-mediated phagocytosis by HD11 cells was found to be induced by allantoic fluid, which is present in inactivated IBV and NDV antigens of poultry vaccines. Nitric oxide production was not induced by allantoic fluid, as demonstrated by the absence of nitric oxide after exposure to IBV and NDV antigens containing allantoic fluid. In this exploratory study, these two assays were able to show different immunopotentiating properties of the vaccines and hence may be used in the future as complementary assays to test for immunostimulatory properties of vaccines. Allantoic fluid might function as an inherent adjuvant and facilitate the immune response, although this remains to be confirmed with in vivo experiments. Finally, the assays used in this study may be applied in the future as quality control tests for inactivated poultry vaccines. Future studies will be needed to address the capacity of these cell-based assays to evaluate vaccine batch-to-batch consistency and detect non-conforming batches, as compared to the animal-based quality control tests that are currently in place as gold standards. Obviously, the capacity of inactivated poultry vaccines to induce antigen-specific immunity requires additional assessments, like antigen quantification or stability, to ensure vaccine quality. The nitric oxide production and phagocytosis assays with HD11 cells might contribute to future efforts to replace current in vivo vaccine batch-release quality tests for in vitro alternatives.

## 5. Conclusions

In this exploratory study we demonstrated that inactivated poultry vaccines are able to activate chicken innate immune cells in vitro. Stimulation of the chicken macrophage-like cell line HD11 with the inactivated poultry vaccines resulted in the production of nitric oxide due the presence of mineral oil adjuvant. Furthermore, inactivated poultry vaccines enhanced Fc receptor-mediated phagocytosis due to the presence of allantoic fluid in the vaccine antigen preparations. Taken together, these two in vitro assays were able to show different immunopotentiating properties of the vaccines and hence may be used in the future as complementary assays to test for immunostimulatory properties of vaccines.

## Supplementary Materials

The following are available online at https://www.mdpi.com/2076-393X/8/2/332/s1, Figure S1: A major part of the beads used for the phagocytosis assay were coupled to IgY, Figure S2: Stimulation with TLR agonists showed little effects on HD11 cell viability, Figure S3: The viability of HD11 cells was affected by various inactivating poultry vaccines to different extents, Video S1: A 3D model of IgY-opsonized beads engulfed by HD11 cells.
