# Supplementary material for: Nitric Oxide Production and Fc Receptor-Mediated Phagocytosis as Functional Readouts of Macrophage Activity upon Stimulation with Inactivated Poultry Vaccines in Vitro
Source: Vaccines (Basel). 2020 Jun 22;8(2):332. doi: 10.3390/vaccines8020332 (PMC7350413; doi:10.3390/vaccines8020332)
Supplement: Supplementary file 1 [file vaccines-08-00332-s001.zip › Supplementary Figures.docx]

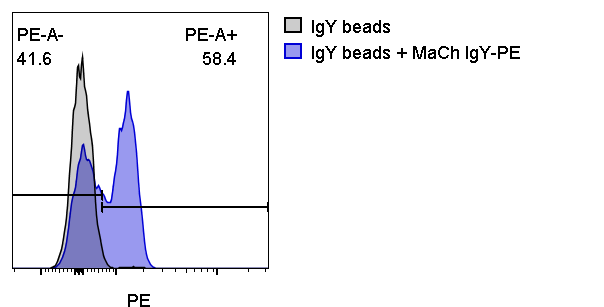


**Supplementary Figure S1** A major part of the beads used for the phagocytosis assay were coupled to IgY. An overlay of histograms shows IgY-coated beads without staining in grey and IgY-coated beads stained with a PE-conjugated mouse anti-chicken IgY in blue.

**Supplementary Figure S2 Stimulation with TLR agonists showed little effects on HD11 cell viability.** The effect of TLR agonists on HD11 cell viability, as determined by zombie aqua fixable/live dead staining, is expressed as the percentage of living cells (%alive). HD11 cells were stimulated with 300 ng/ml LPS, 500 ng/ml CpG, 10 µg/ml R848, 10 ng/ml Pam3CSK4 (Pam), 5 µg/ml zymosan (Zymo), or left unstimulated (Unst). The data comprise three independent replicates performed *in triplo*. Error bars represent the SEM. The experimental groups were tested for statistically significant differences in viability between stimulated and unstimulated groups using a Kruskal-Wallis test and Dunn’s multiple comparisons test. Statistical significance is indicated by * *p* < 0.05, ** *p* < 0.01 and *** *p* < 0.001.

**Supplementary Figure S3 The viability of HD11 cells was affected by various inactivating poultry vaccines to different extents.** The effect of inactivated w/o vaccines on HD11 cell viability, as determined by zombie aqua fixable/live dead staining, is expressed as the percentage of living cells (%alive). HD11 cells were stimulated with a monovalent vaccine (IBV) from company B, bivalent vaccines (IBV + NDV) from companies A, B, and C, and a trivalent vaccine (IBV + NDV + EDSV) from company A. The data comprise three independent replicates performed *in duplo*. Error bars represent the SEM. The experimental groups were tested for statistically significant differences in viability between stimulated and unstimulated groups using a Kruskal-Wallis test and Dunn’s multiple comparisons test. Statistical significance is indicated by * *p* < 0.05, ** *p* < 0.01 and *** *p* < 0.001.
